# Cost-effectiveness of degarelix versus LHRH agonists in prostate cancer: a systematic review

**DOI:** 10.3389/frhs.2026.1608532

**Published:** 2026-03-23

**Authors:** Wei Wang, Sisi Li, Wenxuan Liu, Xiaoyan You, Yang Liu, Xianying Wang

**Affiliations:** 1 Department of Urology, Hebei Medical University Third Hospital, Shijiazhuang, China; 2 School of Pharmacy, Hebei Medical University, Shijiazhuang, China; 3 Department of Pharmacy, Hebei Medical University Third Hospital, Shijiazhuang, China

**Keywords:** degarelix, goserelin, leuprorelin, prostate cancer, systematic review, triptorelin

## Abstract

**Objective:**

The escalating incidence of prostate cancer poses a significant global public health challenge. Optimal utilization of resources is crucial for the effective deployment of funds among the diverse and emerging treatment options for managing prostate cancer. This systematic review aims to offer insights and serve as a reference for pharmacoeconomic studies related to the use of degarelix and luteinizing hormone-releasing hormone (LHRH) agonists in the treatment of prostate cancer.

**Methods:**

We conducted a comprehensive search in databases including Embase, PubMed, the Cochrane Library, CNKI, Web of Science, Scopus, and the Tufts CEA Registry to identify cost-effectiveness studies on the use of degarelix and LHRH agonists in the treatment of prostate cancer, spanning from the inception of these databases up to December 30, 2025. Two independent reviewers sequentially examined titles, abstracts, and full-text articles, applying predefined inclusion and exclusion criteria to select studies for data extraction. Any disagreements were resolved through discussion until a consensus was reached. The quality of the included studies was evaluated using the Quality of Health Economic Studies and Consolidated Health Economic Evaluation Reporting Standards. Relevant data were then summarized and comparatively analyzed, focusing on aspects such as the model framework, model parameters, and uncertainty analysis.

**Results:**

A total of 13 studies were ultimately incorporated, with an overall high quality but significant methodological variations among them. Five studies compared degarelix with leuprorelin, goserelin, or triptorelin; four compared triptorelin to goserelin or leuprorelin; one study evaluated leuprorelin acetate in a 6-month depot formulation vs. a 3-month depot; two compared leuprorelin to goserelin and triptorelin; and one study assessed radiotherapy vs. radiotherapy plus goserelin. Eight studies employed the Markov model, with time horizons spanning from 1 year to 30 years. The majority of the studies (*n* = 7) conducted cost-effectiveness analyses, and most were based in developed countries (*n* = 7). Degarelix was deemed cost-effective in the United States, United Kingdom, and China. Additionally, 6-month depot LHRH agonists were found to be more cost-effective than their monthly or 3-monthly counterparts.

**Conclusion:**

From a societal perspective, the evidence suggests that degarelix may be a cost-effective option for patients with prostate cancer. All assessments of LHRH agonists are of high quality. Among the three LHRH agonists evaluated, the 6-month depot formulation of triptorelin may be a cost-effective option in certain settings. In clinical practice, the evaluation of a drug should comprehensively consider its efficacy, adverse effects, cost-effectiveness, and overall patient survival. The evidence is predominantly derived from high-income countries, and thus the conclusions may have limited generalizability to low- and middle-income country settings.

**Systematic Review Registration:**

https://www.crd.york.ac.uk/PROSPERO/recorddashboard, PROSPERO CRD420250653923.

## 1 Introduction

With the rapid aging of the Chinese population, the incidence rate of prostate cancer (PCa) has soared, placing it ninth among male malignant tumors in China (1). In 2022, the incidence rate reached 9.7 per 100,000, while the mortality rate stood at 4.5 per 100,000, ranking seventh among all male malignant tumors (2). Globally, PCa treatment poses a significant financial burden. In 2021, the global age-standardized rates for incidence, mortality, and disability-adjusted life years were 348.8, 177.93 and 2513.84 per 100,100, respectively (3). In the United States, the cost burden attributable to metastatic PCa exceeds $55,000 per person-year among men with employer-sponsored health insurance and $43,000 among those covered by employer-sponsored Medicare supplement plans (4). Similarly, projections indicate that the incidence and mortality of PCa in China are expected to continue increasing over the next decade, reaching an estimated 315,310 new cases and 81,540 deaths by 2030 (5).

Androgen-deprivation therapy (ADT) is the primary treatment option for locally advanced or metastatic PCa. Among the non-surgical ADT options, degarelix and luteinizing hormone-releasing hormone (LHRH) agonists, such as goserelin, leuprorelin, and triptorelin, are the most widely used. Although numerous studies (6–8) have investigated the cost-effectiveness of degarelix and LHRH agonists in treating PCa, a comprehensive systematic review of economic evaluations encompassing degarelix and these three LHRH agonists is currently absent. Therefore, it is imperative to conduct a thorough and systematic evaluation and analysis of the existing economic research evidence for degarelix and LHRH agonists. This will enable us to assess their cost-effectiveness for PCa patients and provide valuable insights to support clinical rational drug use and medical insurance decision-making.

## 2 Materials and methods

This systematic review and meta-analysis adhered to the PRISMA (Preferred Reporting Items for Systematic Reviews and Meta-Analyses) guidelines to ensure a high standard of quality (9, 10). No ethics approval or patient consent was necessary for conducting this analysis. The protocol has been registered on the PROSPERO website (Registration number: CRD420250653923).

### 2.1 Data sources and search strategies

We conducted a comprehensive search across seven databases (Embase, PubMed, the Cochrane Library, CNKI, Web of Science, Scopus, and the Tufts CEA Registry) spanning from their inception until December 30, 2025. The search terms employed encompassed “luteinizing hormone-releasing hormone”, “LHRH”, “degarelix”, “leuprorelin”, “goserelin”, “triptorelin”, “prostate cancer”, “prostatic neoplasms”, “cost”, “cost-effectiveness”, and “economic evaluation”.

### 2.2 Selection criteria

Pharmacoeconomic studies were included if: (1) full texts were published in English or Chinese; (2) population includes men with Pca; (3) economic evaluations (including cost-effectiveness, cost-utility, cost-minimization, and cost-benefit analyses); (4) exposure to degarelix, or LHRH agonists (leuprorelin, goserelin, or triptorelin). Exclusion criteria were as follows: review articles, editorials and opinions, letters, research protocols.

### 2.3 Data extraction

Data extraction was carried out utilizing pre-designed tables in Microsoft Excel, adhering to the guidelines outlined in the “Cochrane Handbook” chapter on economic evidence (11). The following information was extracted for each economic study: first author, year of publication, analysis method, perspective, location of study, interventions, incremental cost-effectiveness ratio (ICER), total costs, total QALYs, start age, time-horizon, discount rate, model used, outcomes, and study conclusions. The extracted data from the included studies were then analyzed through narrative synthesis.

### 2.4 Assessment of reporting quality

The quality of the pharmacoeconomic studies was evaluated using the Quality of Health Economic Studies (QHES) and a 28-item checklist derived from the Consolidated Health Economic Evaluation Reporting Standards (CHEERS) checklist (12). Each study's quality was assessed based on the responses to the checklist questions, which were coded as “yes” (fully reported and awarded 1 point), “no” (not reported and awarded 0 points), or “partly” (partially reported and awarded 0.5 points). Subsequently, the studies were categorized into four quality tiers: those scoring above 75% were deemed to have “good quality” reporting, those scoring between 50% and 74% were classified as “moderate quality”, those with scores ranging from 25% to 49% were considered “low quality”, and those scoring below 25% were labeled as “very low quality”. Two independent reviewers assessed the ratings, and any disagreements were discussed and resolved.

### 2.5 Data analysis

We conducted a descriptive analysis of the results obtained from the included pharmacoeconomic studies.

## 3 Results

### 3.1 Search results

**Figure 1:**
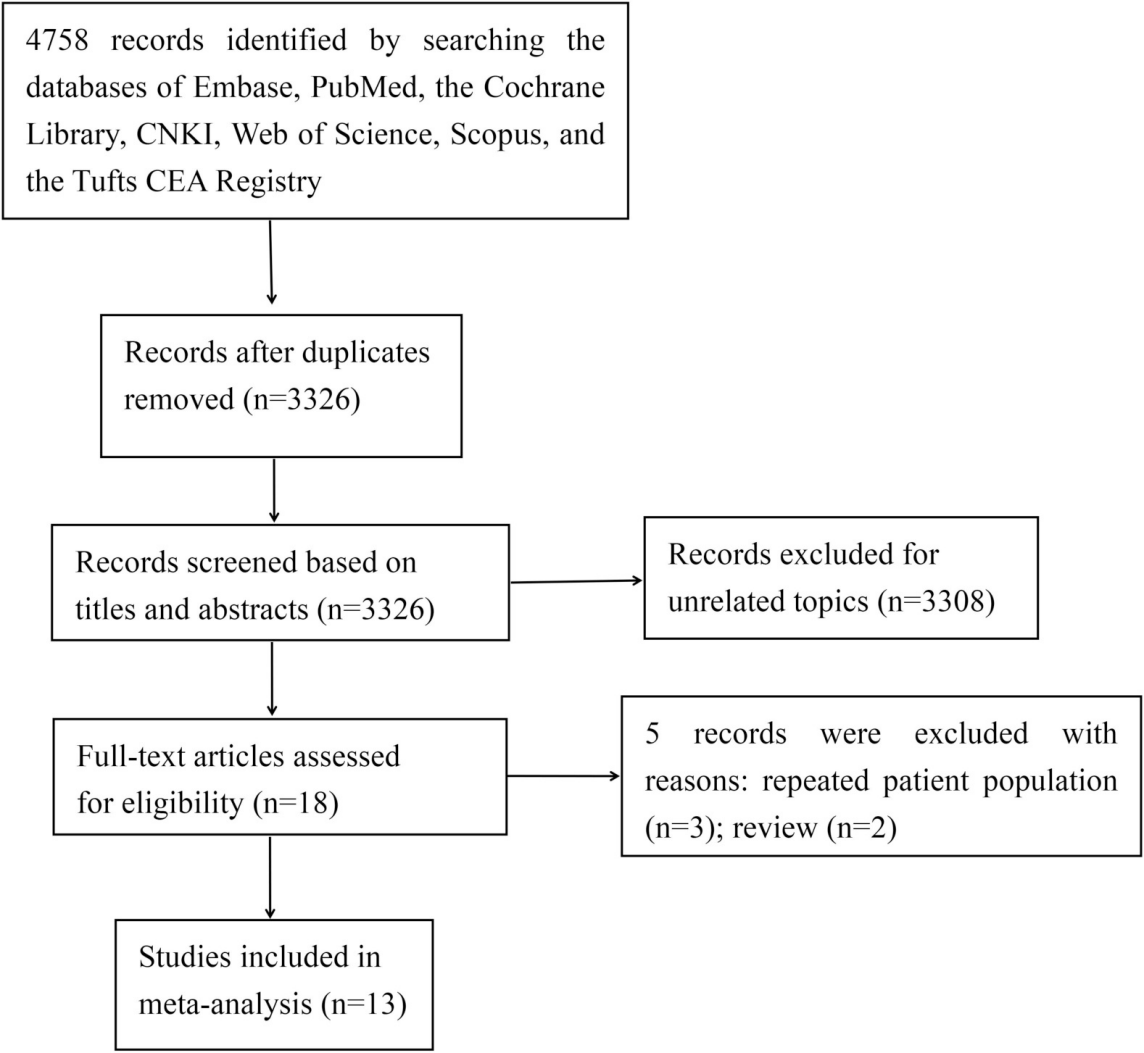
The selection process of the included studies.

Figure 1 outlines the preferred reporting items for systematic reviews. Our literature search yielded a total of 4,578 eligible studies. Following the removal of duplicates, we screened 3,326 abstracts. Among these, 3,308 articles were excluded, leaving 18 full articles to be assessed for eligibility. Ultimately, 13 studies (6–8, 13–22) met the inclusion criteria and were incorporated into this systematic review.

### 3.2 Characteristics of included studies

Table 1 summarizes the characteristics of the included articles. The majority of the studies hailed from China (*n* = 4), while three originated from the United Kingdom, one from Japan, one from Italy, one from the United States, one from France, one from Turkey, and one from Iran. The studies spanned from 2001 to 2024, with a mean/median start age ranging from 68 to 74.6 years. Most studies were conducted from the perspective of a national health service, health insurance system, or healthcare system. Five studies compared degarelix with leuprorelin, goserelin, or triptorelin; four studies contrasted triptorelin with goserelin or leuprorelin; one study evaluated a 6-month depot formulation of leuprorelin acetate vs. a 3-month depot; two compared leuprorelin to goserelin and triptorelin; and one study assessed radiotherapy vs. radiotherapy plus goserelin.

**Table 1:** Summary of included studies.

Author (year)	Analysis	Perspective	Country	Interventions	ICERs	Total Costs	Total Costs exchanged in 2025	Total QALYs	NPV	Start age (years)	Time-horizon (years)	Discount rate	Model	Outcomes	Sensitivity analysis	Study conclusions	CHEERS score
Goto 2017	CMA	Societal	Japan	Leuprorelin acetate 6-month depot vs. leuprorelin acetate 3-month depot	–	–	–	–	JPY 27,265	NA	1	NA	NA	Costs	Subgroup analysis	Leuprorelin acetate 6-month depot has advantage monetary value compared with leuprorelin acetate 3-month depot	85.71%
Hatoum 2013	CEA	Payers	USA	Degarelix vs. leuprolide	$245/QALY	Degarelix $37,174; Leuprolide $36,991	Degarelix $53,001; Leuprolide $52,740	Degarelix 4.203 QALYs; Leuprolide 3.455 QALYs	–	72	20	3.0%	Markov model	Costs, Utility values, QALY, ICER	One-way and probabilistic sensitivity	Degarelix provides a cost-effective treatment for ADT among patients with locally advanced prostate cancer.	83.93%
Iannazzo 2011	CEA	Italian National Health Service	Italy	Leuprorelin 11.25 mg, leuprorelin 22.5 mg, goserelin, triptorelin, buserelin	Vs. Leuprorelin 11.25 mg, -€16,575.66/life year; vs goserelin,-€13,099.01/life year; vs triptorelin,-€53,274.27/life year; buserelin,€140,400/life year	Leuprorelin22.5 mg, €13,981.21; Leuprorelin11.25 mg, €15,113.88; Goserelin, €16,579.18; Triptorelin,€15,934.6; Buserelin, €14,546.25	Leuprorelin22.5 mg, €21,148; Leuprorelin11.25 mg, €22,861; Goserelin, €25,077; Triptorelin,€24,103; Buserelin, €22,003	Leuprorelin 22.5 mg, 5.03 life years; Leuprorelin11.25 mg, 4.96 life years; Goserelin,4.83 life years; Triptorelin,4.99 life years; Buserelin,5.03 life years	–	74.6	5	3.5%	Markov model	Costs, Utility values, QALY, ICER	Probabilistic sensitivity analysis	Leuprorelin 22.5 mg was the most cost-effective treatment of the available depot formulation LHRH agonists.	89.29%
Lee 2014	CUA	UK National Health Service	United Kingdom	Degarelix vs. leuprorelin	-£18,165/QALY	Degarelix, £19,289; leuprorelin 11.25 mg, £22,922	Degarelix, £26,700; leuprorelin 11.25 mg, £31,729	Degarelix, 3.79 QALYs; leuprorelin 11.25 mg, 3.58 QALYs	–	72	20	3.5%	Markov model	Costs, Utility values, QALY, ICER	Deterministic sensitivity analysis and probabilistic sensitivity analysis, scenario analyses	Degarelix is likely to be cost-effective compared to leuprorelin plus anti-androgen flare cover in the first-line treatment of advanced hormone-dependent prostate cancer.	87.50%
Lu 2011	CEA	UK National Health Service	United Kingdom	Degarelix vs triptorelin plus short-term antiandrogen treatment	£59 000/QALY	Degarelix, £3,883; triptorelin+anti-androgen, £3,125	Degarelix, £5,873; triptorelin+anti-androgen, £4,727	Degarelix, 2.4548 QALYs; triptorelin+anti-androgen, 2.4419 QALYs	–	70	10	3.5%	Markov model	Costs, Utility values, QALY, ICER	Probabilistic sensitivity analysis, one-way and multi-way sensitivity analyses	Degarelix is unlikely to be cost-effective compared to triptorelin plus short-term antiandrogen in the management of advanced prostate cancer.	75.00%
Neymark 2001	CEA	French health insurance system	French	Radiotherapy plus goserelin vs. radiotherapy	−11,981 FF/life year	Radiotherapy plus goserelin 58,300 FF; radiotherapy, 71,000 FF	Radiotherapy plus goserelin 118,512 FF; radiotherapy, 144,328 FF	Radiotherapy plus goserelin, 7.05 life years; radiotherapy, 5.99 life years	–	NA	11.2	3.0%	NA	Costs, ICER	Probabilistic sensitivity analysis	Radiotherapy plus goserelin should be considered a cost-effective option compared with radiotherapy alone for locally advanced prostate cancer patients.	91.07%
Yan 2022	CUA	Chinese healthcare system	China	Degarelix vs. leuprorelin	112,674 yuan/QALY	Degarelix 566,226 yuan; Leuprorelin 489,693 yuan	Degarelix 618,730 yuan; Leuprorelin 535,101 yuan	Degarelix 5.19 QALYs; Leuprorelin 4.51 QALYs	–	68	30	5.0%	Markov model	Costs, Utility values, QALY, ICER	Probabilistic sensitivity analysis, scenario analyses, one-way sensitivity analysis	Compared to leuprorelin, degarelix for prostate cancer treatment is cost-effective.	82.14%
Jiang 2022	CEA	Chinese healthcare system	China	Triptorelin vs. goserelin or leuprorelin	Triptorelin 3M vs goserelin 3M, 41,950 yuan/QALY; triptorelin 3M vs leuprorelin 3M, 6,515 yuan/QALY; triptorelin 6M vs goserelin 3M, ‘−30,156 yuan/QALY; triptorelin 6M vs leuprorelin 3M, ‘−82 091 yuan/QALY	Triptorelin 3M, 51,514 yuan; goserelin 3M, 45, 361 yuan; leuprorelin 3M, 50,696 yuan; triptorelin 6M, 50,696 yuan	Triptorelin 3M, 56,291 yuan; goserelin 3M, 49,567 yuan; leuprorelin 3M, 55,397 yuan; triptorelin 6M, 55,397 yuan	Triptorelin 3M, 1.39 QALYs; goserelin 3M, 1.25 QALYs; leuprorelin 3M, 1.27 QALYs; triptorelin 6M, 1.38 QALYs	–	NA	2	NA	Markov model	Costs, Utility values, QALY, ICER	Probabilistic sensitivity analysis, one-way sensitivity analysis	Compared with currently marketed LHRH agonists, triptorelin is a cost-effective strategy in the treatment of locally advanced or metastatic prostate cancer.	78.57%
Xuan 2019	CEA	Chinese health insurance system	China	Degarelix vs. goserelin or leuprorelin	Degarelix vs. goserelin, 42,061 yuan/QALY; degarelix vs. leuprorelin, 80,803.30 yuan/QALY	–	–	–	–	68	10	NA	Markov model	Costs, Utility values, QALY, ICER	Probabilistic sensitivity analysis, one-way sensitivity analysis	Degarelix is cost-effective in a number of scenarios compared to goserelin and leuprorelin in the treatment of prostate cancer in China.	75%
Chen 2024	CMA	Societal	China	6-monthly formulation triptorelin vs 1-monthly or 3-monthly GnRHa therapy	–	–	–	–	–	NA	1	NA	NA	Costs	Univariate sensitivity analysis	Compared to current 1-monthly and 3-monthly formulations, the 6-monthly GnRHa can reduce the total burden associated with prostate cancer treatment.	75.00%
Rezaee 2024	CEA	Societal	Iran	Triptorelin vs. goserelin vs leuprolide	Leuprolide vs goserelin, $15,618.95/QALY; triptorelin vs goserelin, $25,396.73/QALY	Goserelin, $13,539.13; leuprolide, $18,124.75; triptorelin, 26 006.92	Goserelin, $13,945; leuprolide, $18,668; triptorelin, $26,787	Goserelin, 6.365 QALYs; leuprolide, 6.658 QALYs; triptorelin, 6.856 QALYs	–	NA	20	5.80%	Markov model	Costs, Utility values, QALY, ICER	Probabilistic sensitivity analysis, one-way sensitivity analysis	Goserelin was the most effective and cost-effective strategy vs. 2 other options	82.14%
Cornford 2023	CMA	Societal	United Kingdom	Monthly or 3-monthly GnRHa therapy vs 6-monthly formulation	–					NA	1	NA	NA	Costs	One-way sensitivity analysis	Switching/transitioning men from monthly or 3-monthly GnRHa therapy to a 6-monthly formulation can reduce NHS cost and capacity pressures and the societal and environmental costs associated with prostate cancer care.	78.57%
ÖZYİĞİT 2020	CEA	Payers	Turkey	Leuprorelin acetate atrigel vs. leuprolide acetate microsphere, goserelin, and triptorelin	Vs. leuprolide acetate microsphere, −18,186.88 TL/LYG; vs goserelin, −10,637.67 TL/LYG; vs triptorelin, −15,308.67 TL/LYG	Leuprorelin acetate atrigel, 17,236.06 TL; leuprolide acetate microsphere, 25,622.10 TL; goserelin, 20,946.85 TL; triptorelin, 25,682.70 TL	Leuprorelin acetate atrigel, 19,981 TL; leuprolide acetate microsphere, 29,703TL; goserelin, 24,283TL; triptorelin, 29,773 TL	Leuprorelin acetate atrigel, 1.02 LYG; leuprolide acetate microsphere, 0.56 LYG; goserelin, 0.67 LYG; triptorelin, 0.47 LYG	Vs. leuprolide acetate microsphere, 31,351.72 TL; vs goserelin, 21,084.83 TL; vs triptorelin, 35,927.32 TL	NA	10	NA	NA	Costs, life year, ICER	Deterministic sensitivity analysis	Leuprorelin acetate Atrigel was found to be clinically more effective and cost-saving than other LHRH agonists in the intermediate- and high-risk groups, regardless of testosterone suppression targets.	83.93%

### 3.3 Quality evaluation of included documents

The Table 2 presents the scoring results of multiple studies using the QHES assessment system. The studies generally achieved high scores (mostly 7–9 points) in areas such as “study objectives”, “perspective of the study”, “estimation of variables”, “analysis of uncertainty”, and “incremental analysis”, indicating a well-developed core economic analysis framework. However, notable variations were observed across studies in categories such as “subgroup analysis”, “analytical methods”, “time horizon”, “measurement of outcomes”, “reliability and validity”, and “sources of funding”. In particular, all studies scored 0 in “subgroup analysis”, suggesting this dimension was generally not adequately addressed. In terms of total scores, the highest was 99 points (e.g., Hatoum 2013, Iannazzo 2011, etc.), while the lowest was 74 points (Goto 2017). Most studies scored above 90 points, reflecting overall high research quality, though there remains room for improvement in certain methodological details and reporting completeness.

**Table 2:** Quality of health economic studies.

Author (year)	Study objectives	Perspective of the study	Estimation of variables	Subgroup analysis	Analysis of uncertainty	Incremental analysis	Analytical methods	Time horizon	Measurement of costs	Measurement of outcomes	Reliability and validity	Model selection	Assumptions and limitations	Potential biases	Conclusions	Sources of funding	Total score
Goto 2017	7	4	8	0	9	6	0	0	8	0	7	8	0	6	8	3	74
Hatoum 2013	7	4	8	0	9	6	5	7	8	6	7	8	7	6	8	3	99
Iannazzo 2011	7	4	8	0	9	6	5	7	8	6	7	8	7	6	8	3	99
Lee 2014	7	4	8	0	9	6	5	7	8	6	7	8	7	0	8	3	93
Lu 2011	7	4	8	0	9	6	5	7	8	6	7	8	7	0	8	3	93
Neymark 2001	7	4	8	0	9	6	5	7	8	6	7	8	7	6	8	3	99
Yan 2022	7	4	8	0	9	6	5	7	8	6	7	8	7	0	8	3	93
Jiang 2022	7	4	8	0	9	6	5	7	8	6	7	8	7	0	8	0	90
Xuan 2019	7	4	8	0	9	6	5	0	8	6	7	8	7	0	8	0	83
Chen 2024	7	4	8	0	9	6	0	0	8	0	7	8	7	0	8	3	75
Rezaee 2024	7	4	8	0	9	6	5	7	8	6	7	8	7	0	8	3	93
Cornford 2023	7	4	8	0	9	6	0	0	8	0	7	8	7	0	8	3	75
ÖZYİĞİT 2020	7	4	8	0	9	6	5	7	8	6	7	8	7	0	8	3	93

Based on the CHEERS checklist, thirteen studies were deemed to be of high quality. Supplementary Table S1 presents the results of the quality assessment for these studies. Nearly all studies adequately addressed the sections of title, abstract, introduction, methods, results, and discussion. However, several items were less adequately addressed, including the health economic analysis plan, characterization of heterogeneity, the impact of patient engagement and those affected by the study, source of funding, and conflicts of interest. Specifically, fewer than half of the studies described item 4 (health economic analysis plan), item 18 (characterizing heterogeneity), item 27 (source of funding), and item 28 (conflicts of interest). This may be partly attributed to variations in journal requirements. Despite these shortcomings, all included studies were still evaluated as being of good quality.

### 3.4 Economic evaluation results

#### 3.4.1 Degarelix vs. leuprorelin, goserelin, or triptorelin

Yan et al. demonstrated that in Pca patients, degarelix exhibited superior efficacy compared to leuprorelin, yielding 5.19 QALYs vs. 4.51 QALYs. However, the cost of degarelix was higher, amounting to 566,266 yuan compared to 489,693 yuan for leuprorelin. The ICER for degarelix in the treatment of Pca was calculated as 112,674 yuan per QALY, equivalent to 1.39 times the per capita gross domestic product (GDP) of China. Notably, this ICER value is below three times the China GDP per capita in 2021. The findings indicate that degarelix is a cost-effective treatment option for Pca when compared to leuprorelin.

Xuan et al. demonstrated that degarelix is more effective but also more costly when compared to goserelin (with an ICER of 42,060.71 yuan per QALY) and leuprorelin (ICER of 80,803.30 yuan/QALY). Importantly, these ICER values are all below the threshold of one times the GDP per capita. Furthermore, degarelix occupies a dominant position when factoring in the integration of a patient assistance program or a current price discount of 15%.

Hatoum et al. showed that degarelix produced 4.203 QALY and total costs of 37,174 dollars, while leuprolide produced 3.455 QALY with total costs of 36,991 dollars. As such, degarelix yielded an ICER of 245 dollars/QALY. Degarelix provides a cost-effective treatment for ADT among patients with locally advanced Pca.

Lee et al. demonstrated that degarelix is not only more effective but also less expensive (dominant) when compared to leuprorelin 11.25 mg. According to their model, treatment with degarelix, as opposed to leuprorelin 11.25 mg, results in cost savings of £3,633 in the intent-to-treat population and £4,310 in the group with PSA levels greater than 20 ng/ml. Additionally, degarelix leads to gains of 0.20 and 0.24 QALYs in these respective populations.

Lu et al. revealed that, in comparison to triptorelin plus antiandrogen, degarelix incurred total and incremental costs of £758 and resulted in an additional 0.0128 QALYs, respectively. In the base-case analysis, the ICER for degarelix vs. triptorelin plus antiandrogen was estimated to be £59,000 per QALY gained. Based on typical cost-effectiveness thresholds employed in the UK, degarelix is unlikely to be considered cost-effective for the management of advanced Pca when compared to triptorelin plus short-term antiandrogen.

It is noteworthy that four out of five studies suggest that degarelix is a cost-effective therapy for treating patients with Pca.

#### 3.4.2 Triptorelin vs. goserelin or leuprorelin

Jiang et al. demonstrated that, in comparison to the goserelin 3M dosage form, patients utilizing the triptorelin 6M dosage form achieved higher QALYs (+0.14 QALYs) along with a total cost reduction of 4,105 yuan. Furthermore, when compared to the leuprorelin 3M dosage form, the triptorelin 6M dosage form allowed Pca patients to attain higher QALYs (+0.11 QALYs), resulting in a total cost savings of 9,440 yuan. Therefore, the 6M dosage form of triptorelin emerges as a dominant strategy in comparison to the two LHRH agonists mentioned.

Chen et al. revealed that, in comparison to monthly or three-monthly gonadotropin-releasing hormone agonist (GnRHa) therapy, 6M GnRHa treatment saved an average of 12.55 h annually for each patient. Despite an increase in the medical cost for the triptorelin 6M dosage, the overall cost decreased, resulting in a total cost reduction of 13,382,951.13 yuan for Pca patients in China.

Rezaee et al. compared triptorelin with goserelin or leuprolide, and their results showed that the mean cost and utility in the goserelin, leuprolide, and triptorelin arms were respectively 13,539.13 dollars and 6.365 QALY, 18,124.75 dollars and 6.658 QALY, and 26,006.92 dollars and 6.856 QALY. The ICER for leuprolide and triptorelin were 15,618.95 dollars and 25,396.73 dollars, respectively, it could be stated that the ICER of these 2 medicines were above the threshold. Goserelin was considered as a cost-effective treatment option.

Cornford et al. demonstrated that six-monthly triptorelin would reduce the estimated overall annual NHS budget by £5,164,296, with nurse administration costs being the primary driver of cost savings in the model.

Three out of four studies indicate that triptorelin is a cost-effective therapy for treating Pca patients.

#### 3.4.3 Leuprorelin acetate 6-month depot vs. leuprorelin acetate 3-month depot

Goto et al. demonstrated that, when compared to the 3-month depot of leuprorelin acetate, the 6-month depot of leuprorelin acetate results in a total cost reduction of JPY 27,265 for Pca patients in Japan. This finding suggests that the 6-month depot of leuprorelin acetate offers a cost-effective treatment option for Pca patients.

#### 3.4.4 Leuprorelin vs. goserelin, triptorelin, or buserelin

Iannazzo et al. found that, when compared to leuprorelin 11.25 mg, goserelin, and triptorelin, the incremental cost-effectiveness analysis outcomes of leuprorelin 22.5 mg were dominant, as it demonstrated greater effectiveness while incurring lower costs. Consequently, leuprorelin 22.5 mg was identified as the most cost-effective treatment among the available depot formulations of LHRH agonists.

ÖZYİĞİT et al. found that, when compared to leuprolide acetate microsphere, goserelin, and triptorelin, leuprorelin acetate atrigel provided cost savings of 8,386.04 Turkish liras (TL), 3,710.79 TL, and 8,446.64 TL, respectively. Leuprorelin acetate atrigel was found to be clinically more effective and cost-saving than other LHRH agonists in the intermediate- and high-risk groups, regardless of testosterone suppression targets.

#### 3.4.5 Radiotherapy vs. radiotherapy plus goserelin

Neymark et al. demonstrated that, when compared to radiotherapy alone, the combination of radiotherapy and goserelin increased the mean survival time by 1.06 years while simultaneously reducing costs for the French health insurance system by an average of 12,700 FF per patient. Based on these findings, it is highly probable that the combination of radiotherapy and goserelin should be regarded as a cost-effective option compared to radiotherapy alone for patients with locally advanced Pca.

### 3.5 Sensitivity analysis results

Among the thirteen studies examined, ten underwent probability sensitivity analysis. Four studies concurrently conducted both one-way sensitivity analysis and probabilistic sensitivity analysis. Additionally, two studies employed scenario analysis, one conducted subgroup analysis, another performed univariate sensitivity analysis, and yet another carried out one-way sensitivity analysis. The results from these analyses were found to be robust. Table 1 provides detailed descriptions of the sensitivity analysis outcomes. The key parameters involved in these sensitivity analyses encompassed study duration, discount rate, timing of treatment switch, and costs associated with complications.

## 4 Discussion

In this systematic review, thirteen studies that examined the cost-effectiveness of degarelix and various LHRH agonists-specifically goserelin, leuprorelin, and triptorelin for the treatment of PCa were identified. Primarily conducted in developed countries and focusing on adult patients, these studies collectively suggest that degarelix may be a more cost-effective option for PCa treatment compared to goserelin, leuprorelin, and triptorelin. Among the three LHRH agonists, the 6-month depot formulation of triptorelin may be the cost-effective strategy.

Pca boasts a longer natural survival period compared to other types of malignancies. Endocrine therapy for Pca has been in practice for over 70 years, and despite the advent of newer therapies, ADT continues to be the cornerstone of its treatment. Studies have demonstrated that endocrine therapy is effective in prolonging the survival of patients; notably, one study reported a median survival time of 7.81 years for Pca patients undergoing endocrine therapy (23). This particular study presents the inaugural pharmacoeconomic evaluation of degarelix and three types LHRH agonists, analyzed from the healthcare system's perspective. It addresses a significant research gap in this domain and provides valuable, evidence-based insights that can inform clinical practice and promote the judicious use of medications.

The role of funders in conducting research is essential to guarantee that readers can accurately identify potential biases. Seven studies (13–16, 18–20) in our analysis were funded by pharmaceutical companies. Additionally, two studies (7, 8) did not reveal their funding sources, although some of their authors were employed by pharmaceutical companies. Meanwhile, two studies (6, 17) received support from national institutes. Potential impacts of industry sponsorship may include, for example, study designs that tend to highlight the advantages of the sponsor's product, the selection of outcome measures that may favor the intervention group, or selective analysis and reporting of results. At the same time, it should be objectively noted that not all industry-sponsored studies are inherently biased, as some still adhere to rigorous methodological standards. We have assessed the methodological quality of such studies using systematic evaluation tools (such as the QHES and CHEERS assessment tool). However, due to the limited number of such studies or inconsistent reporting of results, quantitative synthesis was not feasible, and therefore the potential influence of sponsorship-related bias on the overall conclusions cannot be entirely ruled out.

Lu et al. (17) yields a contradictory conclusions for the following reasons: (a) the country/region assessed and its drug pricing. The study explicitly states that if the price of degarelix were reduced by 10%, the ICER would drop sharply from £59,000 to £31,000 per QALY. If the price were reduced by 30%, degarelix would become the more cost-effective option. This highlights the central role of the UK market price in 2011 in the study's conclusion; (b) lower or no consideration of the risk of initial treatment-related adverse events. The model assumes that among patients who experience spinal cord compression, the proportion of severe (paralysis-causing) vs. mild cases is split 50%/50%. This proportion is based entirely on local clinician opinion and lacks data support. Sensitivity analysis shows this parameter is a key driver of the results; (c) use of different comparator regimens (e.g., different LHRHa or antiandrogen drugs).

The study conducted by Rezaee et al. adopts a societal perspective, encompassing not only direct medical costs but also direct non-medical costs and indirect costs. The results indicate that the higher “other costs” and direct non-medical costs in the triptorelin group were the primary drivers of its elevated total cost. This fundamentally differs from many studies that focus solely on the healthcare system perspective, concentrating primarily on drug pricing and hospitalization expenses. The study employs a willingness-to-pay threshold based on Iran's per capita GDP (13,116 international dollars), which is significantly lower than thresholds commonly used by institutions like NICE (e.g., £20,000–£30,000 per QALY). This directly influences the criteria for determining “cost-effectiveness”. Iran's drug pricing system, reimbursement rates, and patient out-of-pocket payment structure differ markedly from those in developed countries. The text mentions that although nearly all patients have health insurance, the high direct treatment costs are still primarily borne by the patients themselves. This highlights that drug pricing and the structure of insurance coverage are key drivers of the cost outcomes.

Although this study employed a qualitative synthesis approach rather than conducting traditional quantitative meta-analysis, this decision was primarily due to significant heterogeneity among the included studies in terms of model structure, cost parameters, and time horizon. The included studies utilized various analytical models, such as cost-effectiveness analysis, cost-minimization analysis, budget impact analysis, or Markov models, with variations in state definitions, transition probabilities, and cycle lengths. The estimation of direct medical costs, direct non-medical costs, and indirect costs across studies relied on localized data from different countries or regions and different years. The time frames of the studies ranged from 1 year to 30 years, affecting the discounting and accumulation of costs and outcomes. Given the heterogeneity described above, conducting direct statistical pooling or effect size aggregation could potentially yield misleading results. Therefore, we make a narrative synthesis to systematically summarize and compare the main findings, key assumptions, and conclusions across the studies.

The majority of studies included in this review were conducted in high-income countries, where the structure of the healthcare system, drug pricing mechanisms, clinical practice norms, willingness-to-pay thresholds (typically $20,000–$30,000 per QALY or higher), and local parameters used in cost-effectiveness assessments are highly context-specific. There are orders-of-magnitude differences between low- and middle-income countries and high-income countries in terms of drug prices, healthcare service costs, per capita income, and official willingness-to-pay thresholds (often based on 1–3 times GDP per capita). Interventions deemed “cost-effective” in high-income countries may impose a disproportionately high economic burden in low- and middle-income countries. Limited healthcare resources, low insurance coverage, high patient out-of-pocket expenses, and varying accessibility to different clinical pathways can significantly alter the actual costs and outcomes of interventions. Variations in disease staging, comorbidities, treatment adherence, and the choice of comparator treatments may affect the comparative efficacy of interventions.

Heterogeneity arises from study perspectives, model types and structures, cost and discounting parameters, and utility values and health state valuations. The included studies adopted varying analytical perspectives, which directly influenced the scope of cost inclusions (such as whether indirect costs or patient out-of-pocket expenses were considered). Studies employed different types of decision-analytic models, with varying assumptions, health state definitions, cycle lengths, and time horizons. The cost data years, currency units, exchange rate conversion methods, and discount rates (ranging from 3% to 5%) differed across studies. The sources of utility values used to calculate QALYs varied (e.g., from clinical trials, literature reviews, or mapping studies), and valuations for similar health states may have differed. Variations existed in the choice of comparator treatments, clinical pathways, and target population characteristics (e.g., disease stage, risk stratification) across studies.

We will explicitly state that precisely due to the significant multidimensional heterogeneity described above, it was neither feasible nor methodologically sound to simply pool numerical results or conduct traditional meta-analyses. The reasons include: Non-Comparability of Outcome Measures: ICERs across studies lacked direct comparability due to differences in cost baselines, effectiveness calculation methods, and threshold criteria. Risk of Misleading Pooled Estimates: Statistically pooling highly heterogeneous studies could produce summary results with limited clinical or policy relevance and potentially obscure important contextual differences. Given these considerations, we opted for a structured descriptive synthesis and qualitative comparison. This approach involved systematically presenting study characteristics, key parameters, and outcomes in tables and narrative form, with a focus on analyzing how heterogeneity sources influence the contextual applicability of conclusions.

This systematic review is not devoid of limitations. Firstly, the inclusion of a limited number of studies and the exclusive focus on published literature may introduce potential publication bias. Secondly, the included cost-effectiveness studies varied widely in terms of country backgrounds and data sources, making it challenging to perform a quantitative synthesis of their results. Thirdly, the findings of the studies in this review were heterogeneous. Fourthly, since most studies were conducted in developed countries, further research is needed to assess the applicability of these findings in low- or middle-income countries. Fifthly, a pooled NPV was not calculated due to variations in the comparator treatments across the groups included in this study. Finally, this study only investigated cost-effectiveness in clinical practice. We should comprehensively consider its efficacy, adverse effects, cost-effectiveness, and overall patient survival in clinical practice. In the future, studies that provide a well-designed, head-to-head cost-effectiveness analyses in diverse settings are necessary, particularly in low- and middle-income country settings.

## Data Availability

The original contributions presented in the study are included in the article/Supplementary Material, further inquiries can be directed to the corresponding author.
