# Supplementary material for: Cost-effectiveness of degarelix versus LHRH agonists in prostate cancer: a systematic review
Source: Front Health Serv. 2026 Mar 23;6:1608532. doi: 10.3389/frhs.2026.1608532 (PMC13050778; doi:10.3389/frhs.2026.1608532)
Supplement: Supplementary file 1 [file Table1.doc]

**TABLE 2 Summary of outcome values from included studies**

| Section/topic | Project | Goto2017 | Hatoum2013 | Iannazzo2011 | Lee2014 | Lu2011 | Neymark2001 | Yan2022 | Jiang2022 | Xuan2019 | Chen2024 | Rezaee2024 | Cornford2023 | ÖZYİĞİT2020 |
| --- | --- | --- | --- | --- | --- | --- | --- | --- | --- | --- | --- | --- | --- | --- |
| Title | Title | Y | Y | Y | Y | Y | Y | Y | Y | Y | Y | Y | Y | Y |
| Abstract | Abstract | Y | P | Y | Y | Y | P | Y | Y | Y | Y | Y | Y | Y |
| Introduction | Background and objectives | Y | Y | Y | Y | Y | Y | Y | Y | Y | Y | Y | Y | Y |
| Methods | Health economic analysis plan | Y | N | N | N | N | N | N | N | N | N | N | N | N |
|  | Study population | Y | Y | Y | Y | Y | Y | Y | Y | Y | Y | Y | Y | Y |
|  | Setting and location | N | Y | Y | Y | Y | Y | Y | Y | Y | Y | Y | Y | Y |
|  | Comparators | N | Y | Y | Y | Y | Y | Y | Y | Y | Y | Y | Y | Y |
|  | Perspective | Y | Y | Y | Y | Y | Y | Y | Y | Y | Y | Y | Y | Y |
|  | Time horizon | N | Y | Y | Y | Y | N | Y | Y | Y | Y | Y | Y | Y |
|  | Discount rate | N | Y | Y | Y | Y | Y | Y | Y | N | N | Y | N | N |
|  | Selection of outcomes | Y | Y | Y | Y | Y | Y | Y | Y | Y | Y | Y | Y | Y |
|  | Measurement of outcomes | Y | Y | Y | Y | Y | Y | Y | Y | Y | Y | Y | Y | Y |
|  | Valuation of outcomes | Y | Y | Y | Y | Y | Y | Y | Y | Y | Y | Y | Y | Y |
|  | Measurement and valuation of resources and costs | Y | Y | Y | Y | Y | Y | Y | Y | Y | Y | Y | Y | Y |
|  | Currency, price date, and conversion | Y | Y | Y | Y | Y | Y | Y | Y | Y | Y | Y | Y | Y |
|  | Rationale and description of model | N | Y | Y | Y | Y | N | Y | Y | Y | Y | Y | Y | Y |
|  | Analytics and assumptions | N | Y | Y | Y | Y | Y | Y | Y | Y | P | Y | Y | Y |
|  | Characterizing heterogeneity | Y | N | N | Y | N | N | N | N | N | N | N | N | N |
|  | Characterizing distributional effects | Y | Y | Y | Y | Y | Y | Y | Y | Y | Y | Y | Y | Y |
|  | Characterizing uncertainty | Y | Y | Y | Y | Y | Y | Y | Y | Y | Y | Y | Y | Y |
|  | Approach to engagement with patients and others affected by the study | Y | P | P | Y | Y | P | Y | Y | Y | P | P | P | P |
| Results | Study parameters | Y | Y | Y | Y | Y | Y | Y | Y | Y | Y | Y | Y | Y |
|  | Summary of main results | Y | Y | Y | Y | Y | Y | Y | Y | Y | Y | Y | Y | Y |
|  | Effect of uncertainty | Y | Y | Y | Y | Y | Y | Y | P | P | Y | Y | Y | Y |
|  | Effect of engagement with patients and others affected by the study | Y | P | P | P | P | P | Y | P | P | P | P | P | P |
| Discussion | Study findings, limitations, generalizability, and current knowledge | Y | Y | Y | Y | P | Y | Y | Y | Y | Y | Y | Y | Y |
|  | Source of funding | P | P | P | P | P | P | P | N | N | P | Y | Y | P |
|  | Conflicts of interest | N | Y | N | N | Y | N | Y | N | N | Y | N | N | Y |
| Overall quality | | Good | Good | Good | Good | Good | Good | Good | Good | Good | Good | Good | Good | Good |
